# A novel metamaterial-based antenna for on-chip applications for the 72.5–81 GHz frequency range

**DOI:** 10.1038/s41598-022-05829-0

**Published:** 2022-02-01

**Authors:** Karen N. Olan-Nuñez, Roberto S. Murphy-Arteaga

**Affiliations:** Electronics Department, Instituto Nacional de Astrofísica, Óptica y Electrónica (INAOE), 72840 Puebla, Mexico

**Keywords:** Electrical and electronic engineering, Materials for devices

## Abstract

In this paper we present a novel metamaterial-based antenna simulated using HFSS. The unit cell parameters were extracted using periodic boundary conditions and wave-port excitation. The metamaterial is magnetically coupled to the CPW line, the induced current in the hexagonal ring gives rise to a field perpendicular to the incident one. The antenna can be modeled by an LC circuit. This design achieves a significant impedance bandwidth of 8.47 GHz (S_11_ = − 10 dB from 72.56 GHz to 81.03 GHz), and a minimum return loss of − 40.79 dB at 76.89 GHz, which clearly indicates good impedance matching to 50Ω. The proposed antenna offers gains from 4.53 to 5.25 dBi, with radiation efficiencies better than 74%. Compactness, simple design layout, a novel design, and good radiation characteristics for this antenna are the main contributions of this work. The antenna can be built on top of a 300 µm thick silicon wafer, for application on HR-SOI-CMOS technology. When compared to other antenna designs for the same frequency band, the proposed antenna achieves very good performance. This design is suitable for the reception stage of long-range automobile radar systems, due to its wide HPBW, as well as E-band applications, such as backhaul systems.

## Introduction

To meet the huge public demand for compact, wireless systems, antennas, beside the other necessary electronic circuitry, must be integrated on the same silicon chip, and thus research on on-chip antennas (AoC) has become a very important field of endeavor in recent years^1–8^, just to mention a few.

On-chip antennas offer full monolithic integration of receivers and transmitters, with great repeatability, size reduction, low power consumption, and a reduction of external interconnections, such as bondwires or solder balls^9^. In fact, AoC have become a very dynamic field of endeavor, as the slew of recently published reports shows, spanning different techniques such as coupling and excitation techniques^1–3^, isolation^4^, circuit design^5^, and the use of metamaterial and metasurface properties^6–8^. Of the many applications that have been addressed by different research groups, one that falls in the 76–81 GHz is vehicular radar^10^. Vehicular radar systems are divided into two major areas, the signal processing and power supply unit; and the RF front-end, which contains the radar transceiver device and one or more TX and RX antennas^11^. In fact, on-chip antennas are good candidates for these systems, mainly due to their compact size, low power consumption and the possibility to fully integrate the RF front-end. It is well known, however, that bulk silicon with typical conductivities in the range 1–10 S/m for standard CMOS processes leads to very poor antenna performance, e.g., typical antenna gains of − 10 dBi, due to substrate losses^12^.

Over the past few years, in order to improve the gain, directivity, and radiation efficiency, while overcoming the limitations of silicon substrates and maintaining reduced size, different types of metamaterials have been proposed, such as Artificial Magnetic Conductors, AMC; High Impedance Surfaces, HIS; Electromagnetic Band-Gap structures, EBG; Double Negative Materials, DNG; Zeroth Order Resonators, ZOR; and various types of metasurfaces^13–20^. In other works, external resonators^21^, or lenses are used^22,23^, micromachining is performed around and below the antenna^24^, the doping profile around the antenna is tailored^12^, its position is optimized^25^, reflectors are employed^26^, and high resistivity (HR) substrates are used^27,28^.

Notwithstanding, the majority of on-chip antenna developments have been made on SOI (Silicon-On-Insulator) substrates with HR silicon, but achieving antenna gains in the range of − 3 to 3 dBi. Such low gain values are appropriate for short-range communications, up to one meter; typical applications are the high-data rate transfer and synchronization between smart wireless devices (smart-phone, laptop, external hard drives) using a wireless USB-like connection^12^.

In this paper, we present a novel antenna design based on metamaterial properties that operates in the millimeter wave regime. This design resembles the center of a flower with its petals, and thus we refer to it as a “Flower Metamaterial Antenna”. Unlike classic and traditional antennas, this one is based on a new metamaterial design to operate from 75 to 81 GHz on a HR silicon wafer, and it is excited by proximity with a coplanar waveguide (CPW), covering the spectrum for long-range automotive radars^10^, attaining higher gains to those obtained with SOI technology, and achieving good radiation efficiency.

## Flower-metamaterial antenna design

**Figure 1:**
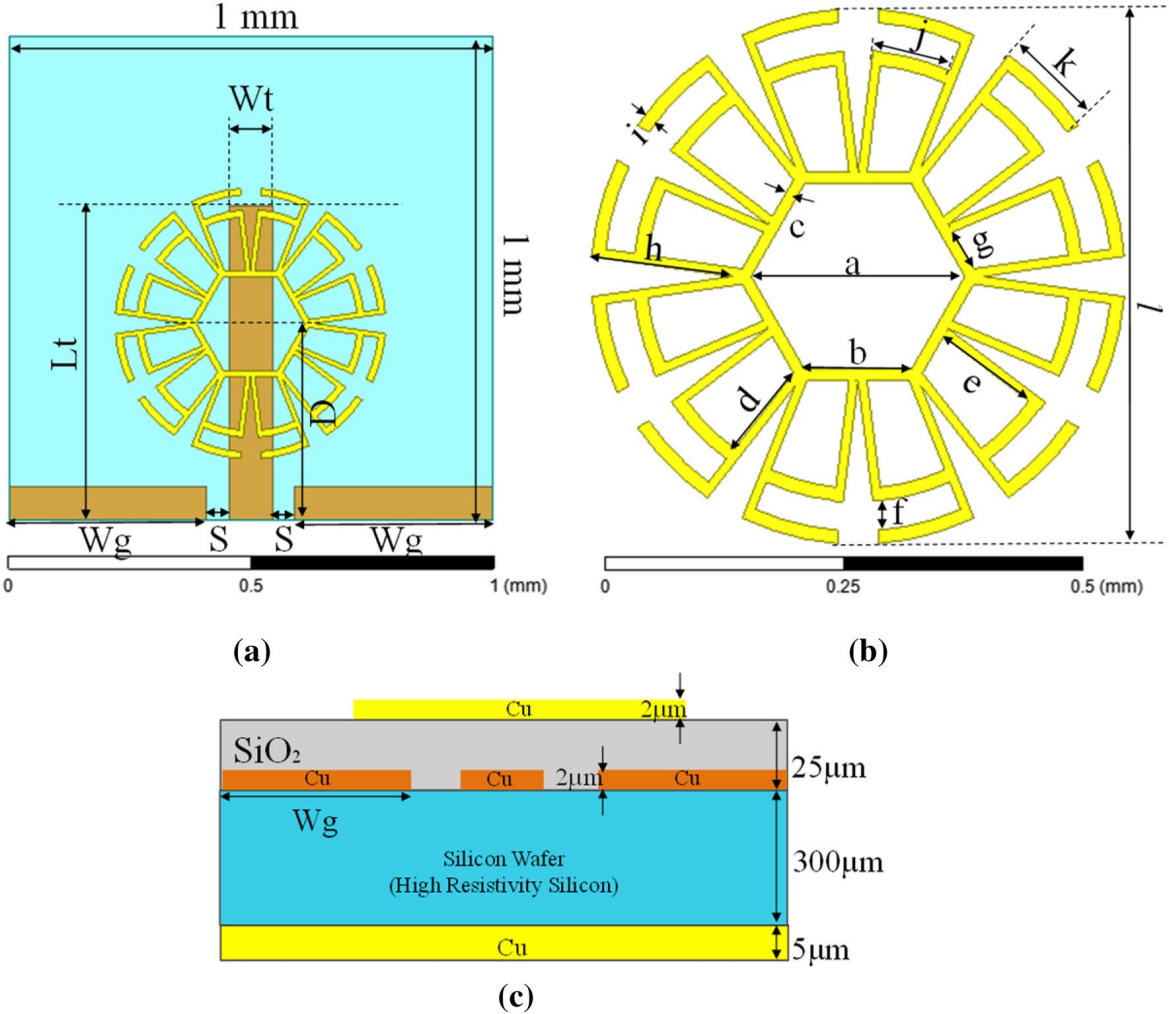
Top view of proposed antenna (**a**) flower-metamaterial antenna and feed line (CPW), (**b**) flower-metamaterial design and (**c**) cross-sectional view of proposed antenna.

The top view of the proposed antenna is shown in Fig. 1a,b. The CPW line used to excite the metamaterial is on a higher metal layer above a thin layer of silicon dioxide. To match the antenna’s input impedance, the width of the feed line (W_t_) is calculated at 90 µm, and the gap between the feed and the ground line on either side (S) is fixed as 45 µm. This CPW feed is highly preferred over a microstrip line in on-chip antenna design since it exhibits lower losses when these lines are deposited directly on high resistivity silicon substrates and are less sensitive to bulk parameter variations such as changes in carrier concentration^27^.

The design parameters for the proposed antenna were parametrically optimized using a full-wave simulator to obtain the desired results, which are listed in Table 1.

**Table 1:** Design parameters.

Parameter	Value (µm)	Parameter	Value (µm)	Parameter	Value (µm)
*a*	224	*g*	44	*D*	400
*b*	112	*h*	140	*Lt*	650
*c*	10	*i*	14	*Wg*	410
*d*	99	*j*	78	*S*	45
*e*	110	*k*	100	*Wt*	90
*f*	29	*l*	560		

Figure 1c depicts a cross sectional view of the proposed structure. A 300 µm thick high resistivity silicon wafer (ρ ≥ 5 kΩ cm, $\tan\delta$ = 0.05 and ${\varepsilon }_{r}$ = 11.8) was used as the substrate. The metamaterial is made of a 2 µm thick copper (Cu) layer. In between the substrate and the radiating structure, there is an insulating layer, namely SiO_2_ (${\varepsilon }_{r}$ = 3.9 and $\tan\delta$ = 0.001) with a thickness of 25 µm, and the feed line (CPW) is placed 23 µm away from the radiating structure in a metal layer embedded in a SiO_2_ layer. Besides, a 5 µm thick metal layer is used as a reflector on the back side.

The Flower-Metamaterial structure was previously designed with the full-wave simulator without the feed line to ensure it behaves as a metamaterial structure. The design was performed following the methodology proposed in^29^, and some details are presented in “Methods” section.

**Figure 2:**
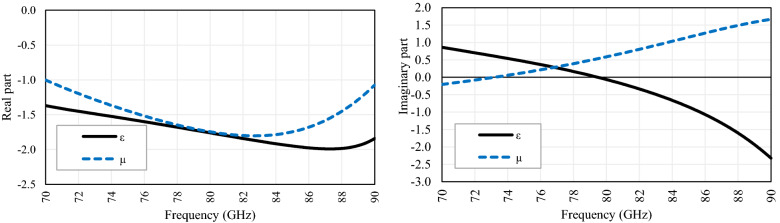
Complex permittivity (ε) and permeability (μ) of proposed flower geometry.

Figure 2 shows the real and imaginary parts of the permittivity and permeability of the design, demonstrating its metamaterial behavior (Left-Handed material) in the frequency band of interest, after a lengthy simulation process.

**Figure 3:**
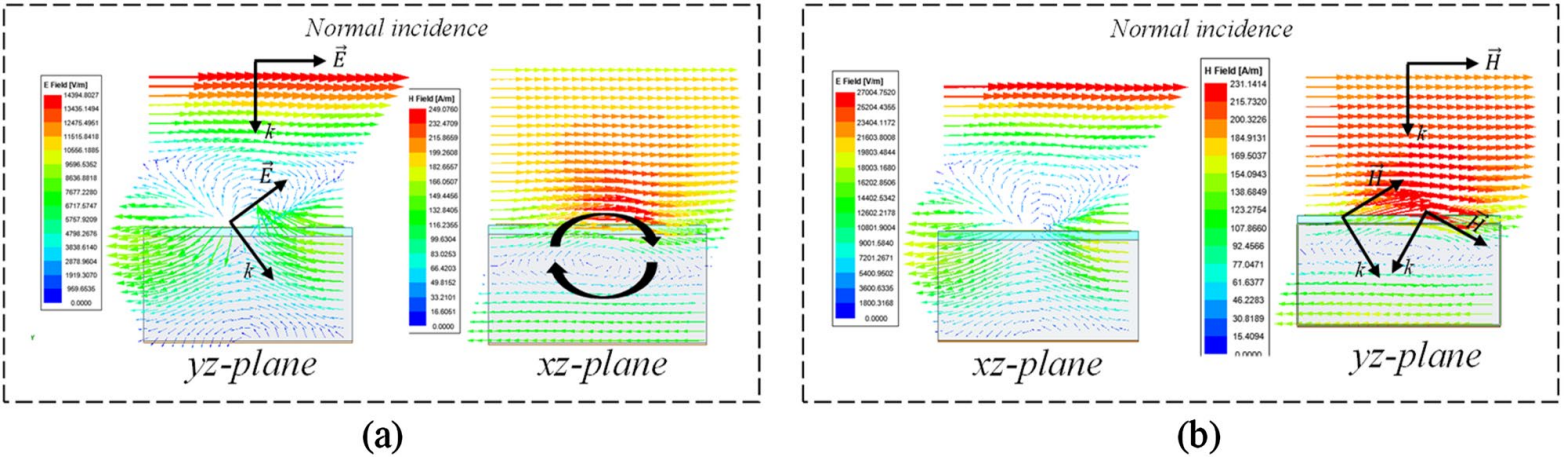
Electrical and magnetic fields for (**a**) TE_00_ mode, and (**b**) TM_00_ mode.

Moreover, when the unit cell is simulated using Floquet ports, the flower metamaterial presents an interesting behavior, which is shown in Fig. 3. From 72 to 81 GHz, the modes supported by the flower are TE_00_ and TM_00_, and other modes (m, n; different from zero) are attenuated (> 30 dB/mm). The flower unit cell changes de propagation direction, curves the direction of electric and magnetic fields, and partially eliminates the magnetic field concentration on the silicon wafer, confining it mostly on and above the flower.

**Figure 4:**
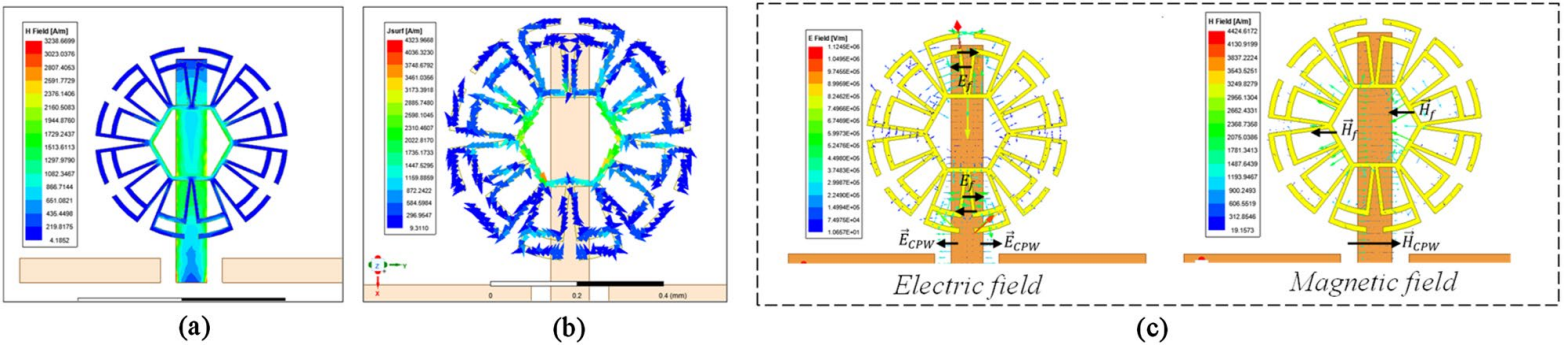
Operation mechanism: (**a**) magnetic coupling, (**b**) induced current, and (**c**) fields throughout the structure.

The operation mechanism is as follows: when the CPW line is positioned below the metamaterial cell, the metamaterial cell is magnetically coupled to the CPW line. The magnetic field lines (of the CPW line) that pass through the hexagonal ring induce a current that gives rise to an electric field in a direction perpendicular to the incident wave. This magnetic coupling, the induced current, and the electric and magnetic fields are shown in Fig. 4.

The design of the proposed unit cell is a lengthy process and many variables play an important role. However, a brief design evolution is presented below with only 5 steps, comparing three important figures of merit considered during the design process.

## Results

This section demonstrates that the proposed design has significant potential for on-chip radar systems, especially for the reception stage, due to its wide HPBW, high gain, small size and ease of fabrication. In the case of the transmitter stage, a moderate to high gain (better than 3 dBi) and a narrow beam are required, and some improvements to the design would be necessary to satisfy them.

**Figure 5:**
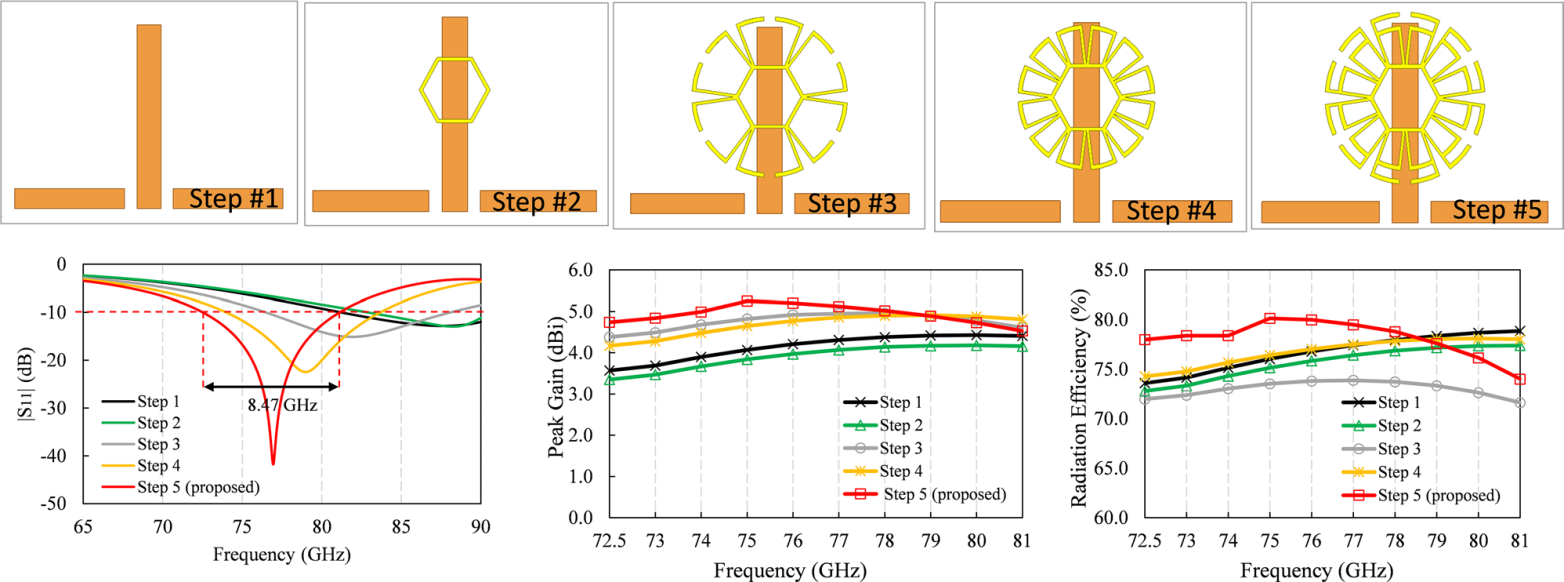
Brief design evolution of the proposed flower metamaterial-based antenna, and comparison of three of the figures of merit versus frequency.

**Figure 6:**
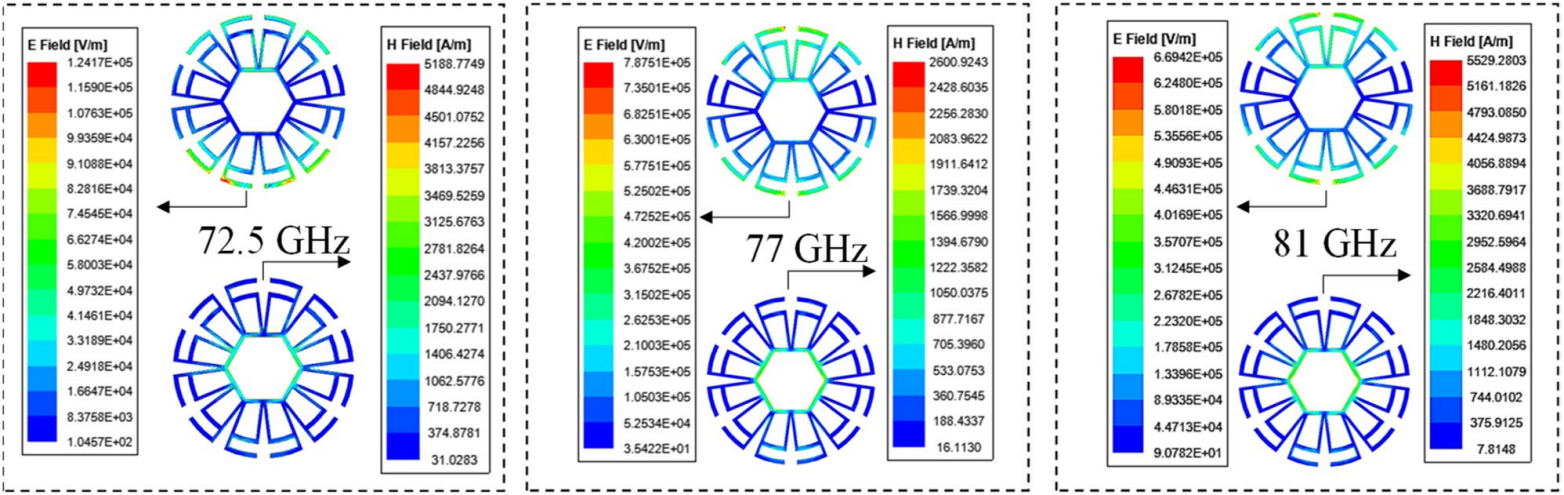
Electric (left side) and magnetic (right side) field magnitude at three frequency points: 72.5 GHz (lower), 77 GHz (central), and 81 GHz (higher).

Figure 5 shows the simulated return loss of the proposed novel flower metamaterial-based antenna and impedance bandwidth (|S_11_| ≤ − 10 dB) of 8.47 GHz, from 72.56 GHz to 81.03 GHz, considering a reference impedance of 50 Ω. The electrical and magnetic planes (H-plane φ = 0° and E-plane φ = 90°) radiation parameters (in magnitude) are presented in Fig. 6, which prove that the design covers the entire frequency band destined for long-range radars (76–81 GHz) and partially the E-band (71–86 GHz).

**Figure 7:**
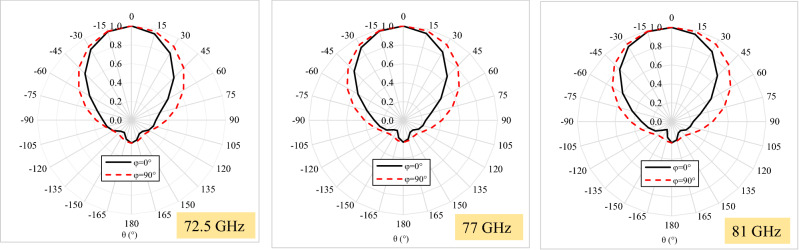
Normalized radiation patterns at three frequency points: 72.5 GHz (lower), 77 GHz (central), and 81 GHz (higher).

The 2D radiation patterns are shown in Fig. 7 for three frequency points (lower, central, and higher), remaining almost unchanged throughout the frequency range from 72.5 to 81 GHz, with only one beam and maintaining symmetry across the bandwidth. The front-back ratio is close to 19 dB, but a higher F/B ratio can be obtained by increasing the reflector plane size.

**Figure 8:**
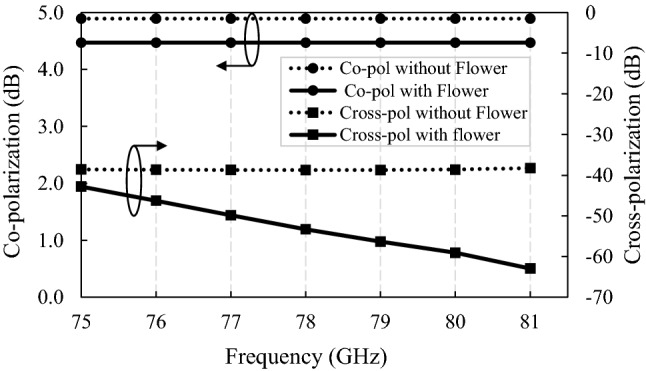
Comparison of cross-polarization and co-polarization versus frequency of the design with and without proposed flower metamaterial.

The comparison of co-polarization and cross-polarization, with and without flower metamaterial, is shown in Fig. 8. This design has cross-polarization values lower than − 30 dB, and co-polarization greater than 4.5 dB, which guarantees that the waves are almost purely linearly polarized to the right, considering the values of axial ratio (AR → ∞) and RHCP-LHCP gains, obtained from the full wave simulator.

Furthermore, the peak gains shown in Fig. 5 show that the proposed design improves gain by 32% at 72.5 GHz, 31.16% at 73 GHz, 27.94% at 74 GHz, 29% at 75 GHz, 23.51% at 76 GHz, 18.79% at 77 GHz, 14.61% at 78 GHz, 10.63% at 79 GHz, 6.8% at 80 GHz and 3.6% at 81 GHz. Likewise, the radiation efficiency is improved from 72.5 GHz to 78 GHz, and from 79 to 81 GHz it decreases slightly, but remains above 74%.

**Figure 9:**
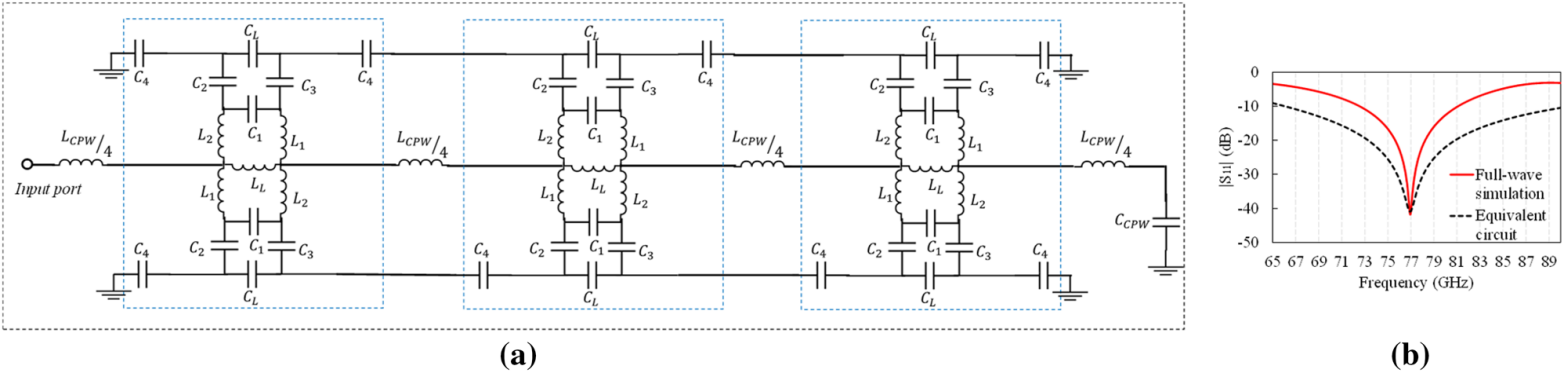
(**a**) Proposed equivalent circuit, and (**b**) comparison of equivalent circuit with full-wave simulation results.

Furthermore, these curves show that the flower material acts as an LC circuit, due to the concentrations of electric and magnetic fields in the design. An equivalent circuit for the metamaterial-based antenna was derived, and it is shown in Fig. 9a. The lumped elements values of the model are: ${L}_{L}=1.56 pH, \; {C}_{L}=2.53 pF, \; {L}_{1}={L}_{2}=10.1 fF, \;  { C}_{1}=20 fF, \; { C}_{2}={C}_{3}=66 pF, \; { C}_{4}=0.1 fF, \; { C}_{cpw}=24.8 fF, \; {L}_{cpw}/4=44.45 pH$. The comparison between model and full-wave simulations is shown in Fig. 9b.

It is noteworthy that this is an original design, which has many advantages over other reported antennas for the same frequency range^13,14,21,22,30,31^, whose characteristics are listed in Table 2.

**Table 2:** Comparison with related works.

Parameter	References								
	^21^	^22^	^13^	^14^	^30^	^31^	^8^	^4^	This work
Substrate and thickness (h)	SiGe	RO3003 and polyphenylene sulfide	Si h = 300 µm	Si h = 200 µm	RO3003 h = 127 µm	RO3010 4350B h = 598 µm	Two layers of polyimide h = 500 µm	Polyimide h = 125 µm	HR-Si h = 300 µm
Total area	~ 3.5 mm^2^	594 mm^2^	1.95 mm^2^	12.96 mm^2^	442.26 mm^2^	> 1500 mm^2^	36 mm^2^	270 mm^2^	1 mm^2^
Design type	Two patches and two quartz glasses	Horn antenna, lens and prism	Monopole antenna, SRR and MIM capacitor	Patch antenna and CSRR	Planar microstrip arrays	L-shaped slots array	CRLH-TL	SIW-loaded patch antenna	Novel flower- metamaterial
Reflection coefficient	− 20 dB	− 45 dB	− 10 dB	− 33.7 dB	< − 20 dB	< − 15 dB	< − 10 dB	–	− 40.79 dB
Bandwidth	N.A.	7 GHz	9 GHz	1 GHz	2 GHz	7 GHz	35 GHz	10 GHz	8.47 GHz
Gain	N.A.	21.5 dBi	1.6 dBi	7.7 dBi	23.3 dBi	16 dBi	8.15 dBi	Max. 12.2 dBi	5.25 dBi
Directivity	N.A.	N.A.	N.A.	N.A.	N.A.	N.A.	–	–	6.55 dBi
Radiation efficiency	50%	N.A.	N.A.	~ 70%	N.A.	N.A.	65.71%	Max. 86%	80.1%
HPBW	N.A.	22°	25°	N.A.	12.2°	9.1°	–	–	126° (φ = 0°) 163° (φ = 90°)
Design frequency	77 GHz	77 GHz	77–87 GHz	77 GHz	77 GHz	79 GHz	0.350–0.385 THz	0.19–0.20 THz	72.5–81 GHz

*N.A.* not available, *SRR* split ring resonator, *CSRR* composite split ring resonator, *CRLH-TL* composite right-left handed transmission line, *SIW* substrate integrated waveguide.

It is important to consider that the designs on ceramic substrates attain a higher gain, since these materials have lower losses than a semiconductor substrate. These designs, however, occupy a very large area and have a narrower bandwidth than our design.

On the other hand^21^, has lower efficiency, occupies a larger area and volume, and is based on a quartz crystal. The design in^13^ has a higher bandwidth and does not occupy a large area, but the gain and coupling at the input are low. The antenna reported by^14^ is approximately 13 times larger than the one presented here, and achieves a gain of just 1.46 times that of the one obtained with the proposed design, in addition to presenting a 1 GHz bandwidth.

Finally, the half power beam width in all the cases is lower than the one obtained in our design, which means that those designs have very fine beams, which are appropriate for the transmission stage, but not for Rx antennas, which require a large field of view^32^.

Additionally, when the proposed design is compared with designs working at THz range^4,8^, this design has lower gain, since both designs^4,8^ use polyimide as substrate; therefore it is to be expected that the gains will be higher, because the substrate has a lower loss coefficient. Compared with^8^ the proposed novel design has higher efficiency, and is 36 times smaller, and compared with^4^, our design is 270 times smaller, even when the operating frequency of our design is lower.

## Discussion

Herein we have presented a novel flower-metamaterial antenna designed to work from 72.5 to 81 GHz. This antenna design, on a HR-Silicon wafer, has medium to high gain, acceptable directivity, good radiation efficiency, wide bandwidth, and compact size, which is ideal for on-chip automobile radar applications, particularly for the reception stage, considering its wide HPBW.

The radiation pattern shows only one beam from 72.5 to 81 GHz. A higher F/B ratio can be obtained by increasing the reflector plane size, and the polarization is almost purely linear, due to good values of cross-polarization and co-polarization in all the range.

The suggested fabrication process for prototyping of the proposed design is as follows: the ground plane, flower metamaterial, and feed line can be of 1–2 μm of copper or aluminum. The thick layer of silicon dioxide can be obtained from wet thermal oxidation process, but also can be replaced with other material, such as polyamide or polyimide, and some dimensions should be adjusted to ensure the impedance bandwidth from 72.5 to 81 GHz.

The new proposed antenna based on the so-called “flower metamaterials” can be integrated into a HR-SOI-CMOS process, in the last layer of the BEOL, that is, because a separation between the excitation line and metamaterial of 23 μm is required, when SiO_2_ is used between both metal layers.

## Methods

All the full-wave simulations were performed using Ansys electromagnetics suite 2021/R1 (High Frequency Structure Simulator, HFSS) (https://www.ansys.com/products/electronics/ansys-hfss).

**Figure 10:**
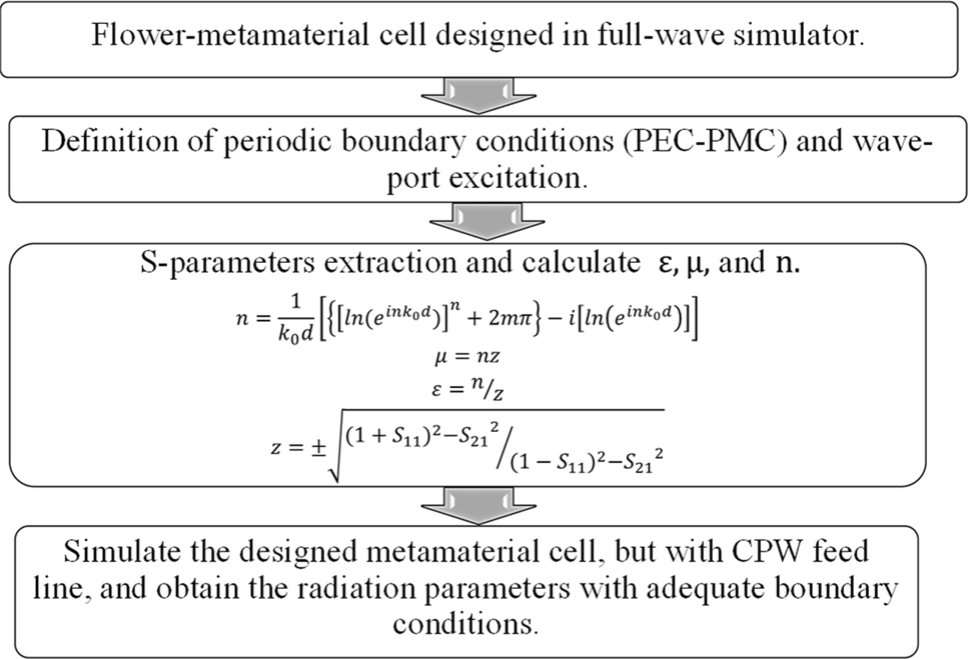
Methodology for design and extraction of parameters of unit cell metamaterial.

For the design and extraction of the parameters of the metamaterial unit cell, the process presented in Fig. 10 was followed. Is an iterative process. Additional simulations were performed with Floquet ports and master–slave conditions to calculate the modes that the flower metamaterial supports, as well as the fields, which are presented in Fig. 3.

For the radiation parameters Ansys is also used, with a lumped port for the excitation with input impedance of 50 Ω and radiation box with dimensions better than ${{\varvec{\lambda}}}_{0} \; (\text{at} \; 80 \; \text{GHz})$. Multiple solution frequencies are used in the simulation to guarantee accuracy across the frequency sweep.

The equivalent circuit was modeled with Advanced Design System (ADS). The proposed equivalent circuit is based on transmission line theory. The three stage shown in Fig. 9a (in blue boxes) represent the flower divided in three parts; ${{\varvec{C}}}_{4}$ represents the capacitance between the *petals* of the *flower shape*; ${{\varvec{L}}}_{{\varvec{L}}}$ and ${{\varvec{C}}}_{{\varvec{L}}}$ are the principal elements of this equivalent circuit, both represents the electromagnetic fields at resonant frequency; and ${{\varvec{L}}}_{{\varvec{c}}{\varvec{p}}{\varvec{w}}}/4$ and ${{\varvec{C}}}_{{\varvec{c}}{\varvec{p}}{\varvec{w}}}$ are the lumped elements of the CPW line. The term ${{\varvec{L}}}_{{\varvec{c}}{\varvec{p}}{\varvec{w}}}/4$ represents the inductance when the flower is magnetically coupled to the transmission line.

## Conclusions

Herein we have presented a novel flower-metamaterial antenna working from 72.5 to 81 GHz. This antenna design over HR-Silicon wafer has medium–high gain (better than 4.5 dBi), good radiation efficiency (higher than 74%), wide impedance bandwidth (8.47 GHz), and compact size (1 mm^2^). Moreover, here we present an equivalent circuit of the novel flower metamaterial- based antenna. The proposed design is suitable for applications of E-band, such as backhaul systems, and automobile radar systems.
